# Analysis of a Regression Model for Creating Surface Microgeometry after Machining Zirconia YML Used for Dental Application

**DOI:** 10.3390/biomimetics9080473

**Published:** 2024-08-05

**Authors:** Ján Duplák, Samuel Mikuláško, Darina Dupláková, Maryna Yeromina, Rastislav Kaščák

**Affiliations:** Department of Automobile and Manufacturing Technologies, Faculty of Manufacturing Technologies, Technical University of Košice, Bayerova 1, 08001 Prešov, Slovakia

**Keywords:** biomaterials, dental implants, milling, zirconium, dental crown

## Abstract

This article focuses on research in the machining of zirconia crowns for dental implants. Its goal is to find the most suitable cutting parameters that significantly affect the final surface roughness of the crowns for dental implants. This study conducts investigations and experiments to specify the cutting parameters that achieve the optimal surface roughness of zirconia crowns for dental implants. The experiments were designed to precisely determine the cutting parameters that influence the surface roughness of zirconia crowns. The results of this study provide important insights for improving the manufacturing process of zirconia crowns with the specified most suitable cutting parameters. This research contributes to the enhancement of zirconia crown manufacturing techniques and the improvement in the quality and effectiveness of dental implants.

## 1. Introduction

For the proper execution of the analysis and subsequent construction of a regression model for the creation of surface microgeometry after machining, it is essential to consider the findings from recent research. The issue of surface roughness of dental crowns needs to be addressed from the perspective of multiple studies, primarily to improve their adaptation and bonding in the context of tooth restoration [1,2]. From the biological point of view, roughness could affect bacterial adhesion, which initiates plague accumulation and causes severe periodontitis or peri-implantitis [3,4]. It is well-known from machining theory and numerous conducted studies [5,6,7,8] that surface roughness is influenced by several factors, including cutting speed, feed rate, and depth of cut. Current research in the field of zirconia dental implant production focuses on optimizing cutting parameters to achieve the desired surface roughness, which is a critical quality attribute. Based on the previously conducted study, the surface roughness value should range between 0.15 and 0.63 µm [9]. However, for the purposes of final finishing (after subsequent polishing or grinding of the milled material) from a medical perspective, the values should be below 0.2 µm, at which point, the surface no longer affects bacterial colonization and adhesion [10]. Studies on the surface roughness of zirconia after milling have shown that cutting speed, depth of cut, and feed rate are the fundamental technological variables affecting roughness. The importance of cutting speed is emphasized in [11,12]. The first study [11] identified speed as the main factor influencing surface roughness during high-speed milling of pre-sintered zirconia in the finishing phase. The second study [12] described a significant impact of speed on surface roughness during the ultrasonic vibration-assisted grinding (UVAG) of sintered zirconia ceramic dental implants. However, these two studies presented conflicting conclusions; the first study highlights the potential for surface improvement at higher speeds, whereas the second study suggests that increased spindle speed may lead to increased surface roughness. This discrepancy underscores the complexity of the machining process and the need for careful optimization of its parameters.

In addition to the aforementioned studies, it is necessary to highlight the current state of the zirconia milling process, one of the primary materials used in dental manufacturing [13,14,15]. The milling process itself is considered the predominant method for producing dental prostheses [16,17,18,19]. However, it is important to emphasize that research has confirmed that not only cutting conditions but also the milling strategies and their combinations can influence the final product. The type of machine used has minimal, if any, impact on the quality of the machined surface [20]. Jing et al. conducted a study comparing self-glazed zirconia machined by CNC milling and produced by additive manufacturing. Their research demonstrated this through the higher fracture strength in zirconia produced by additive manufacturing, attributed to the smaller grain size and fewer voids [21]. Residual stresses were also analyzed in conventional milling and green milling. The results suggest the possibility of eliminating surface voids but not internal ones [22]. Similarly, Chopra et al. [23] emphasized the need to study the relationship between the chemical and physical properties of zirconia oxide concerning its mechanical performance, including the impact of milling. Prescribed surface roughness characteristics are crucial for ensuring the long life of a dental product and resistance to microbes and undesirable organisms [24]. Current research in the technology of zirconia dental product milling using CNC machines with CAD/CAM technology focuses on optimizing cutting parameters to achieve the desired final surface roughness [25,26,27]. Experimental studies on the milling of dental crowns with a focus on final surface roughness involve various methodologies and technologies to optimize the manufacturing process. In 2021, a comparative study was conducted on the production of temporary dental crowns using different 3D printing and milling technologies, highlighting significant differences in accuracy. The research results showed high accuracy values when using milling technology [28]. Modern technologies and strategic approaches bring a significant shift towards digital technologies. Studies compared the accuracy of milling with other manufacturing methods, such as stereolithography (SLA), and found that although the finishing phase significantly affects accuracy, the manufacturing method does not [29,30]. Based on these findings, milling remains the leading technology in dental practice, examined in relation to the final surface of the dental product. In 2014, Lei and Xie identified feed rate as a fundamental parameter influencing surface roughness, with width of cut being the second influencing parameter. Based on their research results, they proposed a linear regression model to predict surface roughness, but in the context of grinding as the final surface finishing process [31].

Previous studies discussed and described research dedicated to the machining of dental materials, such as the Ti6Al4V alloy [32] and Ti3Al2.5V alloy [33]. These studies also focused on optimizing the cutting conditions, such as in the article in [34], where green machining technology was used to optimize the parameters, including X and Y direction cutting speed (mm/min), Z direction cutting speed (mm/min), spindle speed (rpm), path interval (mm), cut-in amount (mm), and finish margin. The issue of optimizing cutting parameters n, v_f_, a_e_, and a_p_ was also addressed in [35], which utilized a polycrystalline diamond tool. In an article presented by us, a combination of materials, machining parameters, and tools used was created but has not yet been published in journals and proceedings. Given the above summary of conducted studies and research, the need for ongoing research is indicated to comprehensively understand the parameters involved in the milling process and their impact on the final surface roughness. By identifying and analyzing these parameters, it is possible to predict and control the entire machining process to achieve the desired outputs. The aim of this study is to determine suitable cutting parameters through the execution of experiments in an external laboratory setting. The result is the identification of cutting parameters that, along with the use of different types of tools, significantly affect the final surface roughness of the machined surface. Based on practical requirements, it is also necessary to identify the most suitable cutting parameters in conjunction with the most appropriate tool among the three selected types. This study expands the research area of material machining for dental applications and provides results for improving the surfaces of dental materials while simultaneously reducing surface roughness after machining, thereby potentially minimizing the need for additional surface treatments.

## 2. Materials and Methods

**Figure 1:**
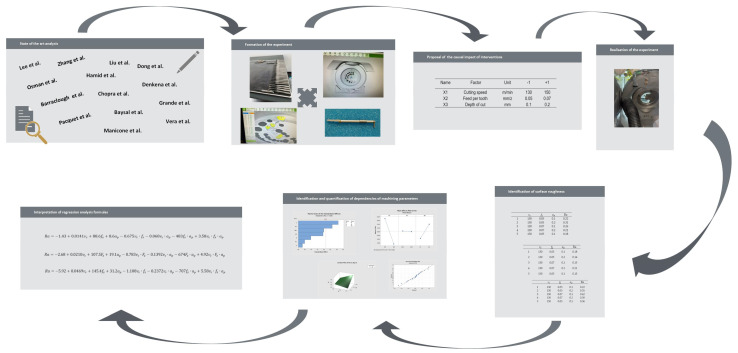
The work methodology diagram.

The aim of this research is to streamline the production process of zirconia dental implants using milling technology through experiments. The experiments will focus on specifying suitable cutting parameters, as the standard production process involves manufacturing implants followed by manual polishing to achieve the desired roughness of Ra 0.2 µm. The outcome of the experiments will be specified cutting parameters that achieve the desired roughness without the need for manual polishing. This experiment was realized in accordance with the Figure 1.

For the experiment, a KATANA™ Zirconia YML (Kuraray Europe GmbH, Hattersheim am Main, Germany) disc was used. This is the most advanced, highly aesthetic zirconia with enhanced graduated translucency (smooth transition from enamel to dentin) and increased durability. The disc consists of 4 layers of zirconia material with varying concentrations of yttria and corresponding mechanical and color properties. The result is a smoothly graduated brightness and translucency effect, without visible transitions and with guaranteed high flexural strength. It is used for manufacturing dental bridges, crowns (frontal and distal sections), substructure constructions for veneering, and inlays/onlays/facets. KATANA™ YML discs can also be processed with rapid sintering (54 min) with minimal waiting times. Optimized material properties improve the final precision and reduce the risk of re-manufacturing [36].

For these experiments, a Datron D5 linear scales machine was used [37]. Several types of cutters with different coating types were also used. The material being machined was a zirconia oxide disc (Table 1). The semi-finished product had a diameter of 98 mm and a thickness of 18 mm. During the experiment, 24 dental crowns were manufactured.

The experiment was prepared based on the design of the experiment method—the full factorial experiment. The focus of this study was to find the most suitable cutting parameters for the production process of dental bridges made of zirconium oxide. The measured output parameter was surface roughness (Ra). The experiments investigated cutting speed, feed rate, and depth of cut, and various tools were used. The results in the experiments were determined using the Ra parameter. For each measurement, 3 combinations of the cutting parameters X1 cutting speed, X2 feed rate, X3 depth of cut were used (Table 2). From the table, it is evident that 3 series of measurements were performed for the same cutting parameters. The individual values were the arithmetic average of 12 measurements conducted on each sample. In each of the three experiments, a different type of tool was used in the machining process. The first tool was a double-sided carbide cutter with a 30° tip angle without coating p.24 code—00781015 [39]. The second tool was a double-sided carbide cutter with a 30° tip angle with X.CEED (AlTiN) coating p.31 code 0078015L [40]. The third tool was a double-sided carbide cutter with a 15° tip angle without coating p.26 code—0068815A [39]. The cutting parameters were designed from the product catalog of the respective tools. 

**Figure 2:**
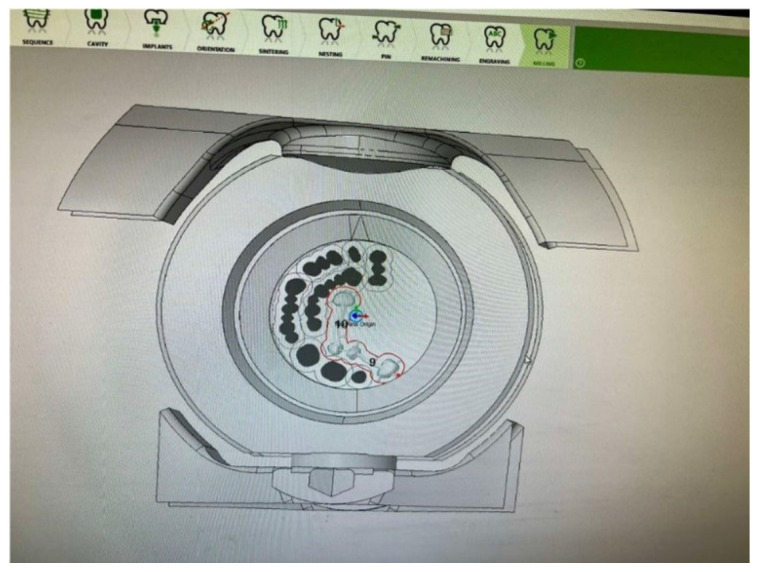
Model identification of the manufacturing area.

In each experiment, the same manufacturing process conditions were used, with the only variation being the choice of tools. The experiment was prepared using the WORKNC Dental software Xpert 5-Axis, where the dental model (Figure 2) to be manufactured was designed [39]. WorkNC Dental is a specialized CAD/CAM software designed for dental milling, enabling the efficient production of highly accurate dental prostheses. The software supports the import of CAD files from various sources, including intraoral and laboratory scanners, accepting formats such as STL and PLY. Users can manually or automatically designate areas of the model for machining, with options for detailed adjustment and editing of these areas.

**Figure 3:**
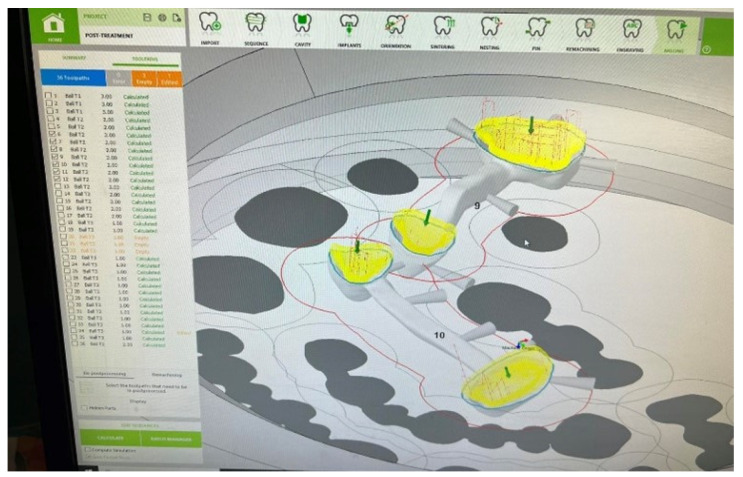
Defining surfaces and calculations for the milling simulation process.

Also, then the code for CNC machining (Figure 3) on the Datron D5 machining center with a spindle characteristic of 1.8 kW; 50,000 RPM; and rotational axis tilting angles A, ±25°, and B, ±45°, was generated. WorkNC Dental allows users to define surfaces and perform calculations for the milling simulation process with high precision. Upon importing a CAD model, the software automatically identifies and analyzes the geometric properties of the model. Subsequently, the software performs the necessary calculations and generates the milling simulation process, enabling the visualization and optimization of the entire milling procedure. This process ensures that the resulting dental restorations are manufactured with the required accuracy and quality.

This software offers a user-friendly interface that enables the milling of complex dental prostheses. It not only automates the milling process but also can be used to automate the implant placement process. It is an open-source CAM software product, meaning it can be used with any standard milling machine. Machining sequences are optimized and dependent on the material, using 4, 3 + 2, or simultaneous 5-axis strategies. The software is primarily designed for metal, ceramic, plastic, and nanocomposite materials. It features 3D machine dynamics and kinematics control. Another advantage is the automatic generation of 5-axis toolpaths for machining. With this software, machining of any type of dental component from any material is possible.

The roughness measurements were conducted in accordance with ISO 4288 (Surface texture: Profile method, 1996), utilizing a cut-off length of 0.8 mm, a measuring length of 4 mm, and a velocity of 0.1 mm/s with a Gaussian filter. Each sample had 12 measurement segments, and the surface was measured directly after machining, without any additional surface treatments.

## 3. Results and Discussion

### 3.1. Evaluation of the First Experiment

The result of the experiment is the parameter of roughness Ra. From Table 3, it is evident that three series of measurements were conducted for the same cutting parameters. The individual values are the arithmetic average of 12 measurements (12 repetitions) performed on each sample. In the first series of measurements, a tool with a 30° tip angle without coating was used in production. In the series of measurements, the cutting speeds were 130 m/min and 150 m/min, the feed per tooth was 0.05 mm/z and 0.07 mm/z, and the depth of cut was 0.1 mm and 0.2 mm.

In the conducted experiments, a significance level of 0.05 was established. This value, α = 0.05, indicates an acceptance of a 5% risk of rejecting the null hypothesis when it is true. If the p-value is less than 0.05, the null hypothesis is rejected, indicating that the observed effect is statistically significant.

In the first experiment, the overall statistical significance of the model was established with a *p*-value of 0.035, indicating that at least one factor or interaction significantly affects the dependent variable Ra. The values for cutting speed and depth of cut are not statistically significant, while the value for feed per tooth is at the borderline of statistical significance. The interactions v_c_·f_z_, f_z_·a_p_, and v_c_·f_z_·a_p_ are also not statistically significant. However, in the conducted experiment, the interaction v_c_·a_p_ is statistically significant. 

**Figure 4:**
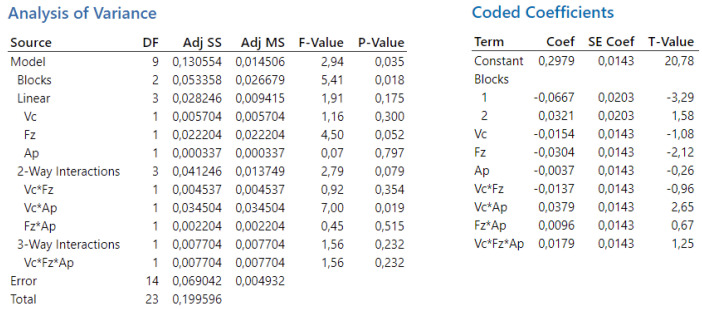
The first experiment-analysis of variance (**left**) and T-values (**right**).

In the conducted analysis of variance, the total number of degrees of freedom (DF) is 23. These degrees of freedom are allocated between the individual components of the model and the error term. For the calculation of t-values, 14 degrees of freedom are attributed to the error component. The results of the analysis are presented in Figure 4.

**Figure 5:**
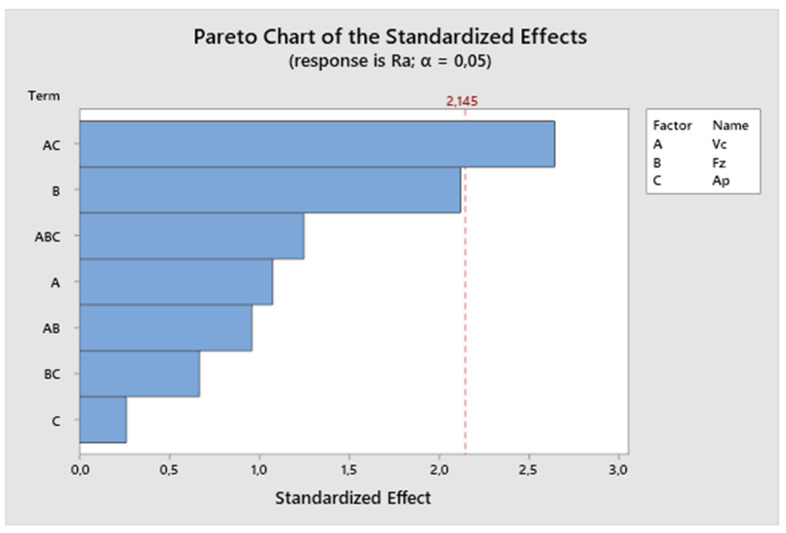
The first experiment—Pareto chart of the standardized effect.

The size of the factors’ impact on the resulting value can be observed from the Pareto chart (Figure 5). The effects are ranked from the largest to the smallest. From the Pareto analysis conducted for the first experiment, it can be concluded that the most significant factor influencing the resulting roughness is the cutting speed in combination with the depth of cut. In this experiment, the feed rate also significantly affects the resulting surface roughness. The depth of cut alone has the least effect on the final surface roughness. 

**Figure 6:**
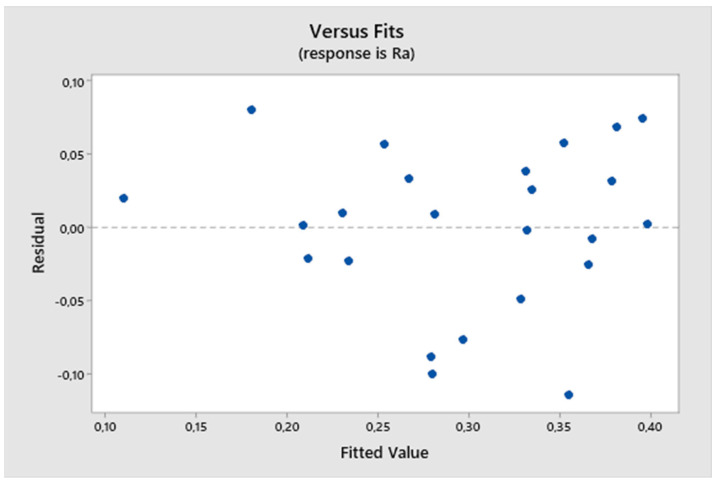
The first experiment-fitted values and residuals.

From the residual plot (Figure 6), it can be seen that it is a model fulfilling the assumptions because the residual deviations are randomly distributed around zero, and the studentized residuals range from −0.2 to 0.2. The residuals do not exhibit heteroscedasticity (they form an irregular cluster), indicating no variance in the values during measurement.

**Figure 7:**
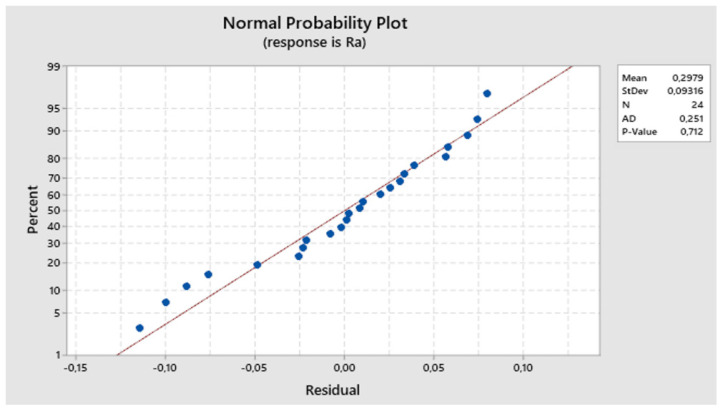
The first experiment-expected values and residuals.

The residuals align around the ideal line, indicating a normal distribution. The normal probability plot of residuals displayed in Figure 7 allows us to accept the hypothesis of residual normality.

After analyzing the variations in measurements, a regression equation was obtained. This equation denotes the surface roughness as a function of the independent factors of cutting speed, feed rate, and depth of cut. The following equation describes the basic regression model:

(1) $$Ra=-1.43+0.0141v_{c}+88.6f_{z}+8.6a_{p}-0.675v_{c}\cdot f_{z}-0.068v_{c}\cdot a_{p}\;-483f_{z}\cdot a_{p}+3.58v_{c}\cdot f_{z}\cdot a_{p}$$

**Figure 8:**
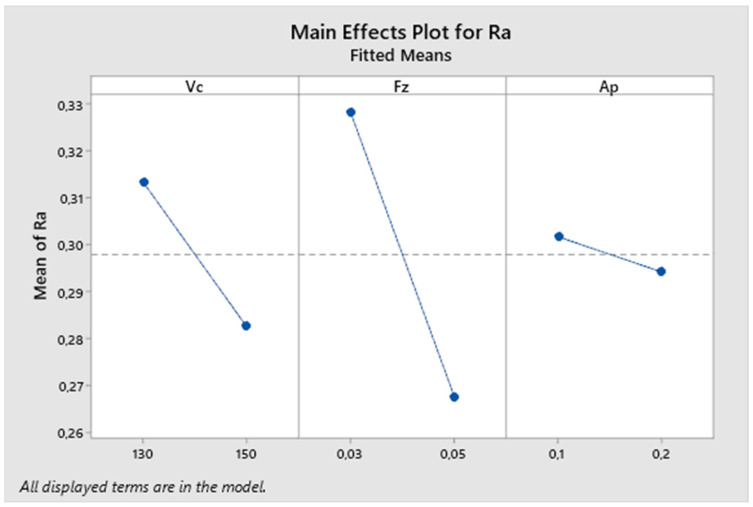
The first experiment-main effects plot for Ra.

The next graph in Figure 8 describes the influence of individual parameters on the resulting surface roughness. From the graph, it can be observed that the feed rate parameter has the greatest impact on the resulting roughness, followed by the cutting speed parameter. The influence of the depth of cut parameter is much smaller in this experiment compared to others.

**Figure 9:**
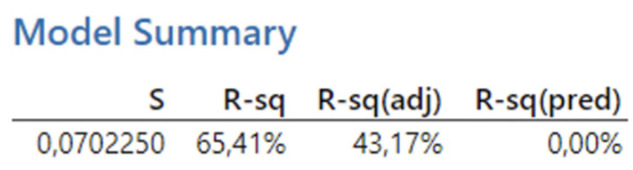
The first experiment-model summary.

Figure 9 presents the reported coefficients of determination for the first experiment. The results obtained from the analysis show a coefficient of determination (R-squared) of 65.41%, which falls within the interval < 50, 80), indicating a high degree of fit. Thus, over 65% of the variability in the dependent variable can be explained by a linear relationship with the predictors. However, after adjusting for the number of predictors, the Adjusted R-squared value decreases significantly to 43.17%, which falls within the interval < 30, 50), indicating a moderate degree of fit. 

Subsequently, through backward regression, the least significant variables were gradually removed from the model to ensure the identification of only statistically significant variables. In the analyzed case, based on the reported *p*-values, the variables with the highest *p*-values were considered for removal. The first variable eliminated was the depth of cut, with a *p*-value of 0.797. Next, the interaction term f_z_·a_p_ was removed, with a *p*-value of 0.515. This was followed by the elimination of v_c_·f_z_ (*p*-value 0.354) and the cutting speed (*p*-value 0.300). Finally, the term v_c_·f_z_·a_p_ was removed with a *p*-value of 0.232. The resulting regression equation includes variables with *p*-values lower than 0.05 f_z_ (*p*-value 0.052, which is borderline but acceptable) and v_c_·a_p_ (*p*-value 0.019). The resulting regression equation after applying backward regression is as follows:

(2) $$Ra=0.2979-0.0304f_{z}+0.0379v_{c}\cdot a_{p}$$

In the following graphs, the influence of different combinations of parameters on the resulting surface roughness can be observed. The most suitable surface roughness was measured on the graph $f_{z}\cdot v_{c}$, where the surface roughness was recorded at around 0.24 µm.

**Figure 10:**
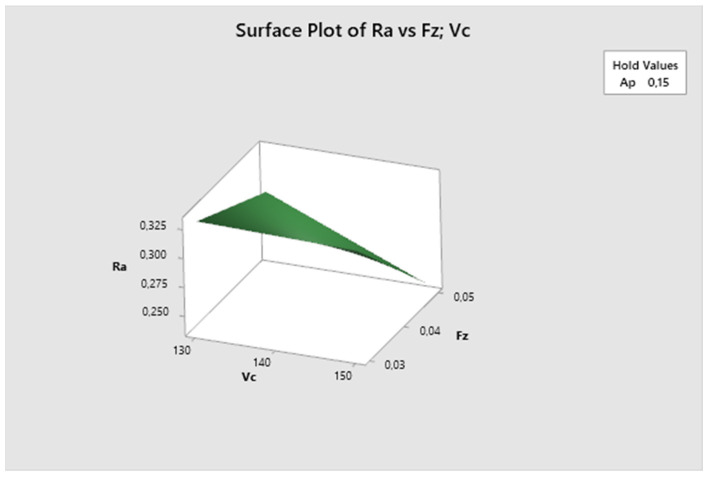
The first experiment-surface plot of Ra—$f_{z}$; $v_{c}$.

The following graph (Figure 10) represents the surface roughness Ra dependency on the combination of two process factors. For each measurement, the third factor is fixed at the middle level. The lowest Ra values are obtained with higher cutting speeds and higher feed rates and increase with decreasing cutting speed. The lowest Ra value is observed at $v_{c}$ 150 m/min and $f_{z}$ 0.05 mm/z. 

**Figure 11:**
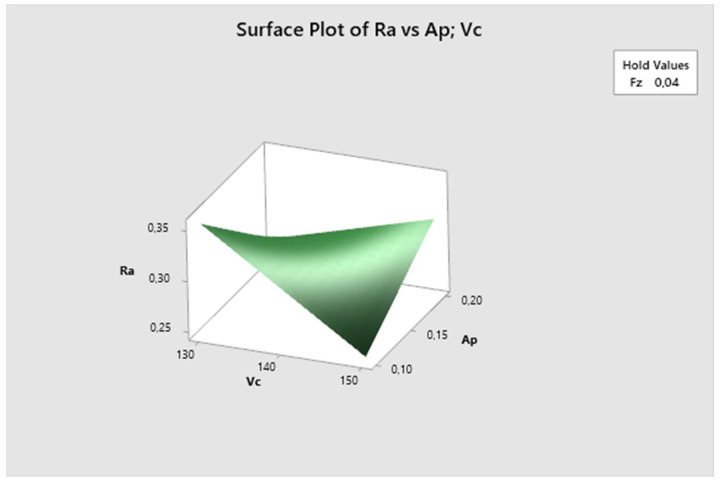
The first experiment-surface plot of Ra—a_p_; v_c_.

In this graph—Figure 11—the feed rate parameter is fixed. Here, it can be seen that the lowest Ra values are obtained with higher cutting speeds and lower depth of cut, increasing with decreasing cutting speed. The lowest Ra value is observed at $v_{c}$ 150 m/min and $a_{p}$ 0.1 mm.

**Figure 12:**
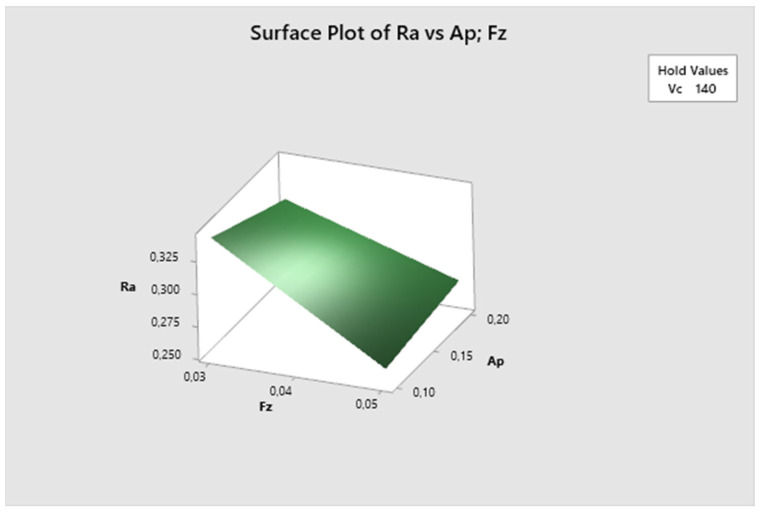
The first experiment-surface plot of Ra—a_p_; $f_{z}$.

The cutting speed parameter is fixed in Figure 12. Here, it can be seen that the lowest Ra values are obtained with higher feed rates and lower depth of cut, increasing with higher depth of cut. The lowest Ra value is observed at $f_{z}$ 0.05 mm/z and $a_{p}$ 0.1 mm.

### 3.2. Evaluation of the Second Experiment

The result of the experiment is the parameter of roughness Ra. From Table 4, it is evident that three series of measurements were conducted for the same cutting parameters. The individual values are the arithmetic average of 12 measurements performed on each sample. In the first series of measurements, a tool with a 30° tip angle with coating X.CEED was used in production. In the series of measurements, the cutting speeds were 130 m/min and 150 m/min, the feed per tooth was 0.05 mm/z and 0.07 mm/z, and the depth of cut was 0.1 mm and 0.2 mm.

In the second experiment, the overall statistical significance of the model was established with a *p*-value of 0.043, indicating that at least one factor or interaction significantly influences the dependent variable Ra. The individual effects of cutting speed, depth of cut, and feed per tooth were found to be not statistically significant. Similarly, the interactions v_c_·f_z_ and f_z_·a_p_ were not statistically significant. However, the interactions v_c_·a_p_ and v_c_·f_z_·a_p_ were found to have a statistically significant effect.

**Figure 13:**
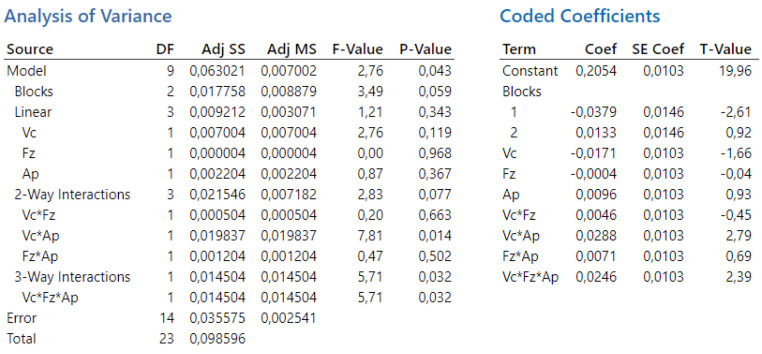
The second experiment-analysis of variance (**left**) and T-values (**right**).

In the conducted analysis of variance, the total number of degrees of freedom (DF) is 23. These degrees of freedom are allocated between the individual components of the model and the error term. For the calculation of t-values, 14 degrees of freedom are attributed to the error component. The results of the analysis are presented in Figure 13.

**Figure 14:**
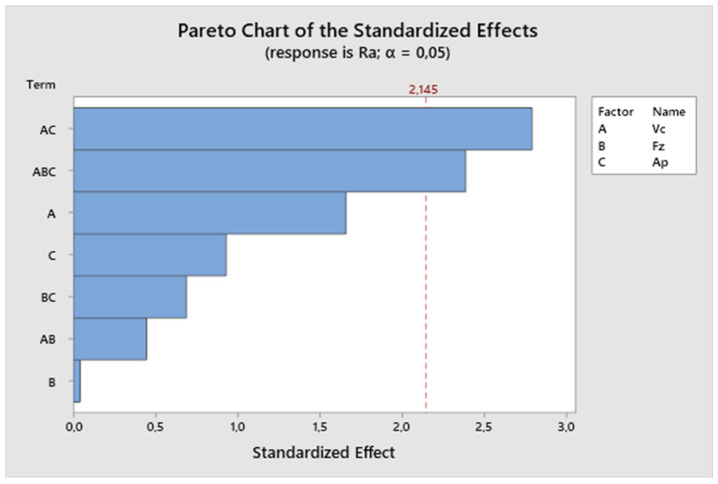
The second experiment-Pareto chart of the standardized effects.

Based on the measured values, a Pareto analysis was conducted. The size of the influence of factors on the resulting value can be seen from the Pareto chart (Figure 14). The effects are ranked from the largest to the smallest. From the Pareto analysis conducted for the second experiment, it can be observed that the most important factor influencing the resulting roughness is the cutting speed. Also, it is evident that the combination of all three parameters significantly affects the final surface roughness. The individual parameter feed rate is negligible in this series of measurements.

**Figure 15:**
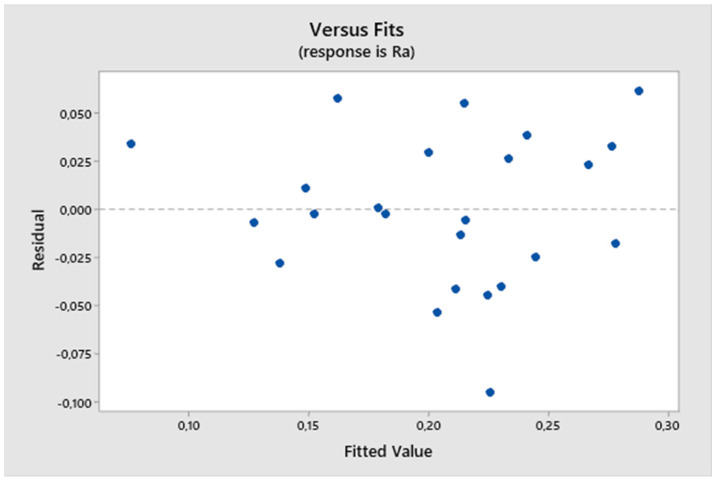
Second experiment-fitted values and residuals.

From the residual plot (Figure 15), it is evident that it meets the assumptions because the residual deviations are randomly distributed around zero, and the studentized residuals range from −0.1 to 0.1. Additionally, the residuals do not exhibit heteroscedasticity (forming an irregular cluster), indicating no variance in values during the measurement.

**Figure 16:**
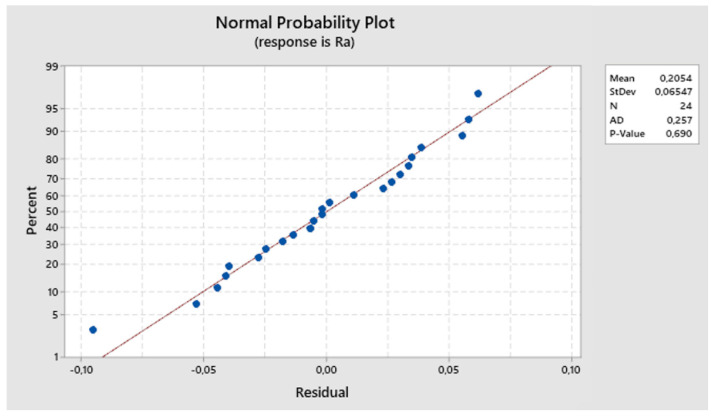
Second experiment-expected values and residuals.

The residuals align around the ideal line, suggesting a normal distribution. The normal probability plot of residuals depicted in Figure 16 allows us to accept the hypothesis of normality concerning the residuals.

Following the analysis of variance measurements, a regression equation was derived. This equation represents surface roughness as a function of independent factors: cutting speed, feed rate, and depth of cut. The following equation describes the basic regression model:

(3) $$Ra=-2.68+0.0210v_{c}+107.5F_{z}+19.1a_{p}-0.783v_{c}\cdot F_{z}-0.1392v_{c}\cdot a_{p}\;-674F_{z}\cdot a_{p}+4.92v_{c}\cdot F_{z}\cdot a_{p}$$

**Figure 17:**
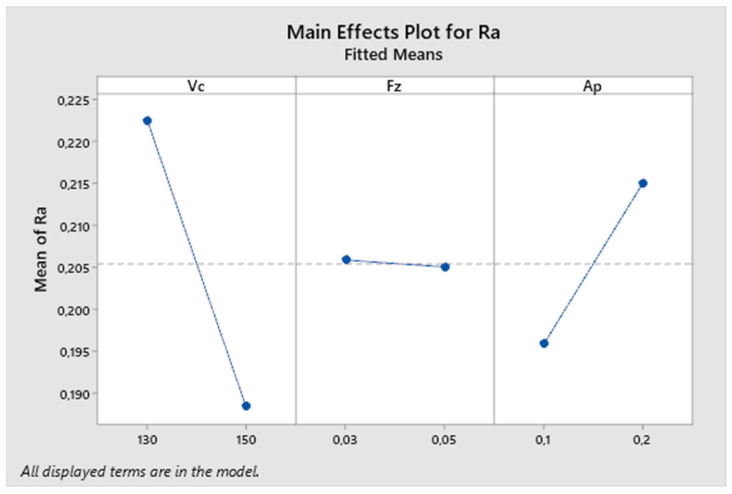
The second experiment-main effects plot for Ra.

In the next graph—Figure 17—the influence of individual parameters on the resulting surface roughness is described. From the graph, it can be noted that the cutting speed parameter has the greatest effect on the resulting roughness, followed by the depth of cut. The influence of the feed rate parameter in this experiment is much smaller compared to others. 

**Figure 18:**
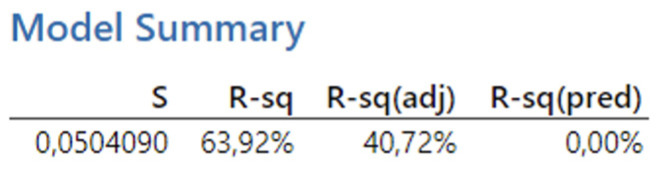
The second experiment-model summary.

Figure 18 presents the reported coefficients of determination for the second experiment. The results obtained from the analysis show a coefficient of determination (R-squared) of 63.92%, which falls within the interval < 50, 80), indicating a high degree of fit. Thus, over 65% of the variability in the dependent variable can be explained by a linear relationship with the predictors. However, after adjusting for the number of predictors, the Adjusted R-squared value decreases significantly to 40.72%, which falls within the interval < 30, 50), indicating a moderate degree of fit.

Subsequently, through backward regression, the least significant variables were gradually removed from the model to ensure the identification of only statistically significant variables, the same as in the first experiment. In the analyzed case, based on the reported *p*-values, the variables with the highest *p*-values were considered for removal. The first variable eliminated was the feed per tooth, with a *p*-value of 0.968. Next, the interaction term v_c_·f_z_ was removed, with a *p*-value of 0.663. This was followed by the elimination of f_z_·a_p_ (*p*-value 0.502), the depth of cut (*p*-value 0.367), and the cutting speed (*p*-value 0.119). Finally, the resulting regression equation includes variables with *p*-values lower than 0.05: v_c_·a_p_ (*p*-value 0.014) and v_c_·f_z_·a_p_ (*p*-value 0.032). The resulting regression equation after applying backward regression is as follows:

(4) $$Ra=0.2054+0.0288v_{c}\cdot a_{p}+0.0246v_{c}\cdot f_{z}\cdot a_{p}$$

On the following graphs, the impact of different combinations of parameters on the resulting surface roughness can be observed. The most suitable surface roughness was observed on the $a_{p}\cdot v_{c}$ graph, where the surface roughness measured approximately 0.15 µm.

**Figure 19:**
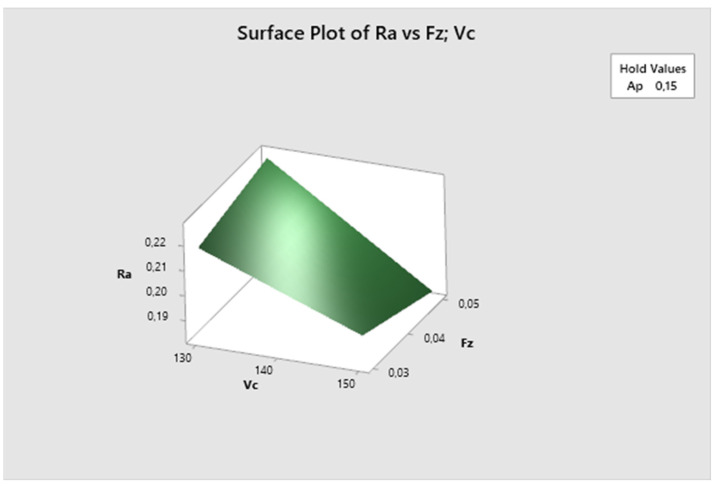
The second experiment-surface plot of Ra—$f_{z}$; $v_{c}$.

The following graph (Figure 19) illustrates the surface roughness Ra dependency on the combination of two process factors. For each measurement, the third factor is fixed at the middle level. The lowest Ra values are obtained with higher cutting speed and higher feed rate, and they increase with decreasing cutting speed. The lowest Ra value is observed at $v_{c}$ 150 m/min and $f_{z}$ 0.05 mm/z.

**Figure 20:**
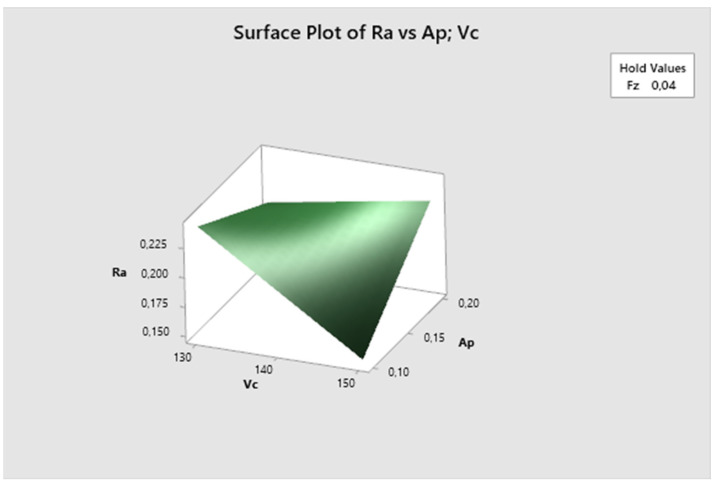
The second experiment-surface plot of Ra—$a_{p}$; $v_{c}$.

Figure 20 demonstrates that the feed rate parameter is fixed, and it is evident that the lowest Ra values are achieved with higher cutting speed and lower depth of cut, increasing with decreasing cutting speed. The lowest Ra value is observed at $v_{c}$ 150 m/min and $a_{p}$ 0.1 mm.

**Figure 21:**
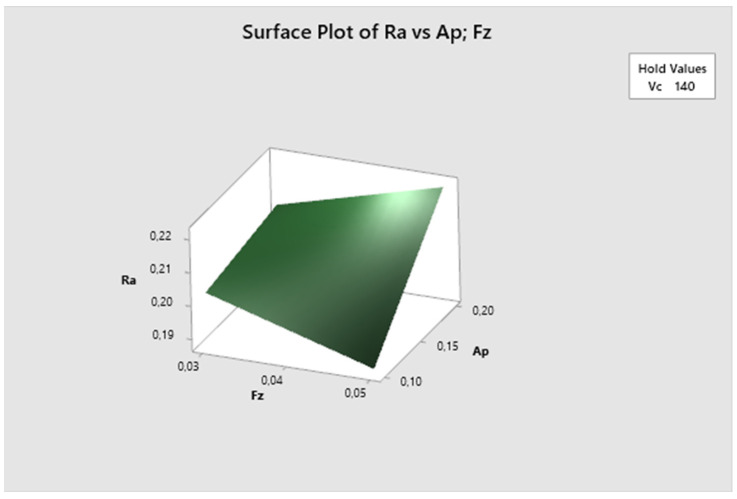
The second experiment-surface plot of Ra—$a_{p}$; $f_{z}$.

The cutting speed parameter is fixed in Figure 21. It is evident that the lowest Ra values are achieved with higher feed rate and lower depth of cut, increasing with higher depth of cut. The lowest Ra value is observed at $f_{z}$ 0.05 mm/z and $a_{p}$ 0.1 mm.

### 3.3. Evaluation of the Third Experiment

The result of the experiment is the parameter of roughness Ra. From Table 5, it is evident that three series of measurements were conducted for the same cutting parameters. The individual values are the arithmetic average of 12 measurements performed on each sample. In the first series of measurements, a tool with a 15° tip angle without coating was used in production. In the series of measurements, the cutting speeds were 130 m/min and 150 m/min, the feed per tooth was 0.05 mm/z and 0.07 mm/z, and the depth of cut was 0.1 mm and 0.2 mm.

In the third experiment, the overall statistical significance of the model is indicated by a *p*-value of 0.034, suggesting that at least one factor or interaction significantly affects the dependent variable Ra. The variables cutting speed and feed per tooth are not statistically significant. The interaction term vc×ap is also not statistically significant, while the interactions v_c_·f_z_ and v_c_·f_z_·a_p_ are on the border of statistical significance. In this experiment, the depth of cut is statistically significant, as is the interaction f_z_·a_p_.

**Figure 22:**
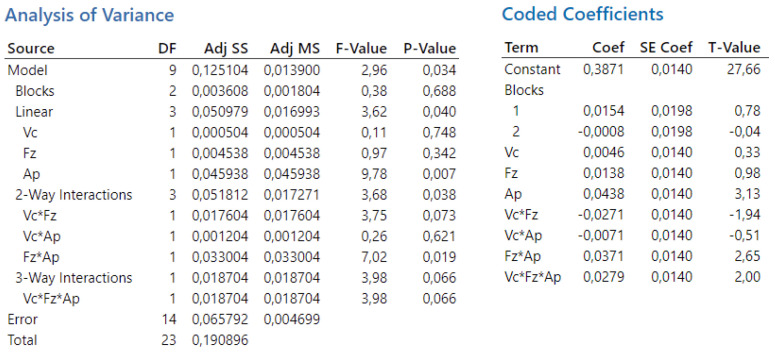
The third experiment—analysis of variance (**left**) and T-values (**right**).

In the conducted analysis of variance, the total number of degrees of freedom (DF) is 23. These degrees of freedom are allocated between the individual components of the model and the error term. For the calculation of t-values, 14 degrees of freedom are attributed to the error component. The results of the analysis are presented in Figure 22.

**Figure 23:**
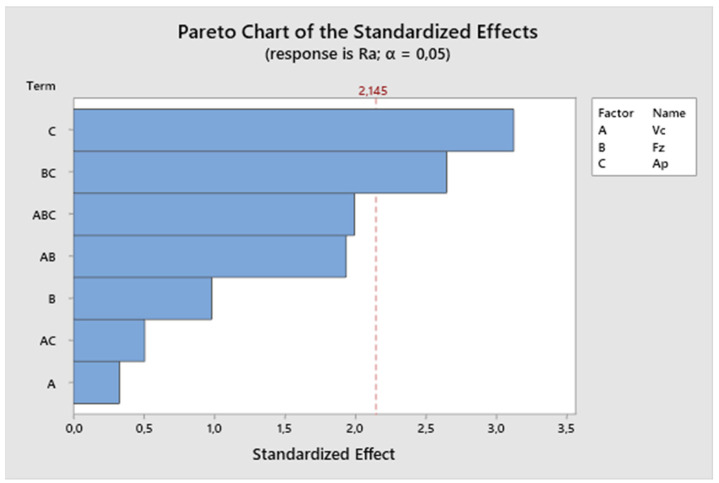
The third experiment-Pareto chart of the standardized effect.

The magnitude of the factors’ influence on the outcome can be observed from the Pareto chart (Figure 23). The effects are ranked from largest to smallest. From the Pareto analysis conducted for the third experiment, it can be concluded that the most significant factor affecting the surface roughness is the depth of cut, combined with the feed rate this time. In this experiment, the cutting speed has much less impact on the surface roughness compared to previous experiments. 

**Figure 24:**
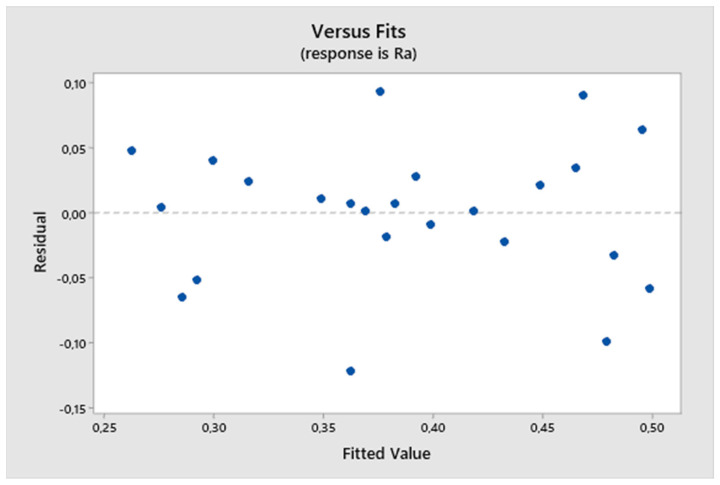
The third experiment-fitted values and residuals.

From the residual plot (Figure 24), it is evident that the model meets the assumptions because the residual deviations are randomly distributed around zero, and the studentized residuals range from −0.2 to 0.2. The residuals do not exhibit heteroscedasticity, indicating that there was no dispersion of values during the measurement.

**Figure 25:**
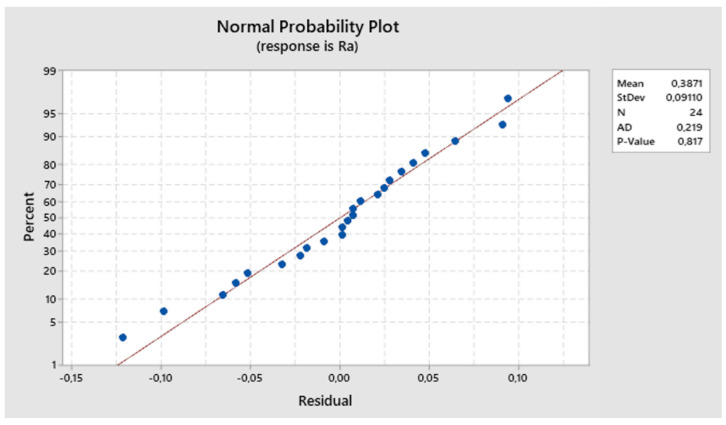
The third experiment-expected values and residuals.

The residuals align around the ideal line, suggesting that they follow a normal distribution. The normal probability plot of residuals, shown in Figure 25, supports accepting the hypothesis of residual normality.

After analyzing the variations in measurements, a regression equation was derived. This equation represents surface roughness as a function of independent factors: cutting speed, feed rate, and depth of cut. The following equation describes the basic regression model:

(5) $$Ra=-5.92+0.0469v_{c}+145.4f_{z}+31.2a_{p}-1.108v_{c}\cdot f_{z}-0.2372v_{c}\cdot a_{p}\;-707f_{z}\cdot a_{p}+5.58v_{c}\cdot f_{z}\cdot a_{p}$$

**Figure 26:**
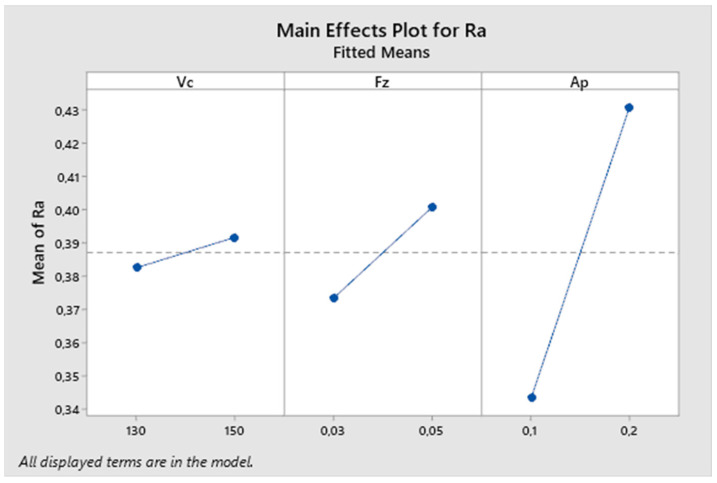
The third experiment-main effects plot for Ra.

In the next graph in Figure 26, the influence of each parameter on the resulting surface roughness is described. From the graph, it is evident that the parameter with the greatest impact on surface roughness is the depth of cut. The influence of the cutting speed and feed rate parameters is much smaller in this experiment compared to others. 

**Figure 27:**
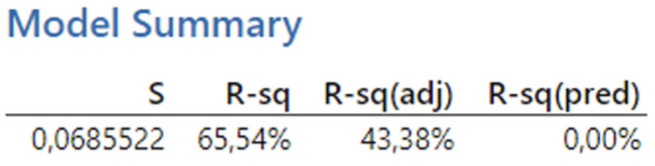
The third experiment-model summary.

Figure 27 presents the reported coefficients of determination for the third experiment. The results obtained from the analysis show a coefficient of determination (R-squared) of 65.54%, which falls within the interval < 50, 80), indicating a high degree of fit. Thus, over 65% of the variability in the dependent variable can be explained by a linear relationship with the predictors. However, after adjusting for the number of predictors, the Adjusted R-squared value decreases significantly to 43.38%, which falls within the interval < 30, 50), indicating a moderate degree of fit.

Subsequently, through backward regression, the least significant variables were gradually removed from the model to ensure the identification of only statistically significant variables, the same as in the first and the second experiments. The first variable eliminated was the velocity of cutting, with a *p*-value of 0.748. Next, the interaction term v_c_·a_p_ was removed, with a *p*-value of 0.621. This was followed by the elimination of feed per tooth (*p*-value 0.342), then the interaction term v_c_·f_z_ (*p*-value 0.073), and the interaction term v_c_·f_z_·a_p_ (*p*-value 0.066). Finally, the resulting regression equation includes variables with *p*-values lower than 0.05: depth of cut (*p*-value 0.007) and f_z_·a_p_ (*p*-value 0.019). The resulting regression equation after applying backward regression is as follows:

(6) $$Ra=0.3871+0.0438a_{p}+0.0371f_{z}\cdot a_{p}$$

In the subsequent graphs, one can observe the impact of different combinations of parameters on the resulting surface roughness. The most suitable surface roughness was measured on the $a_{p}\cdot f_{z}$ graph, where the surface roughness was approximately 0.33 µm.

**Figure 28:**
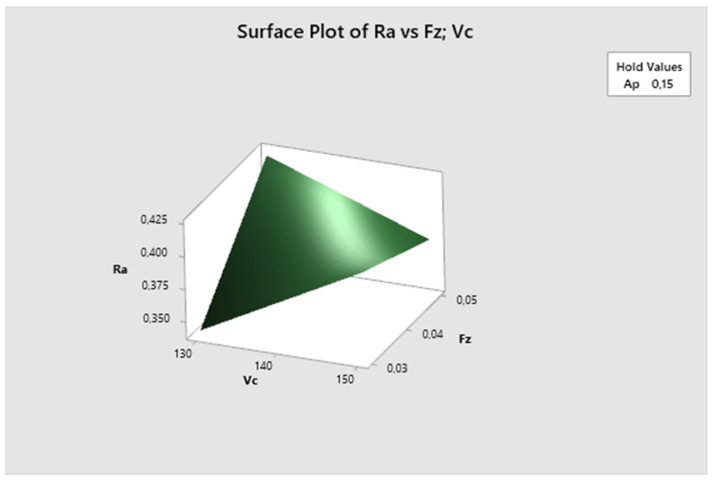
The third experiment-surface plot of Ra—$f_{z}$; $v_{c}$.

The following graph (Figure 28) illustrates the surface roughness Ra dependency on the combination of two process factors. For each measurement, the third factor is fixed at the middle level. The lowest Ra values are obtained with lower cutting speed and lower feed per tooth, and they increase with decreasing cutting speed. The lowest Ra value is observed at $v_{c}$ 130 m/min and $f_{z}$ 0.03 mm/z.

**Figure 29:**
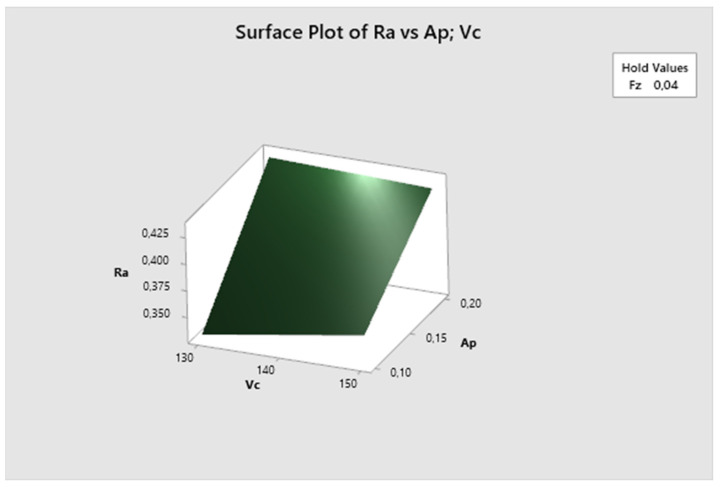
The third experiment-surface plot of Ra—$a_{p}$; $v_{c}$.

The feed per tooth parameter is fixed in Figure 29, and it is evident that the lowest Ra values are achieved with lower cutting speed and shallower depth of cut, increasing with decreasing cutting speed. The lowest Ra value is observed at $v_{c}$ 130 m/min and $a_{p}$ 0.1 mm.

**Figure 30:**
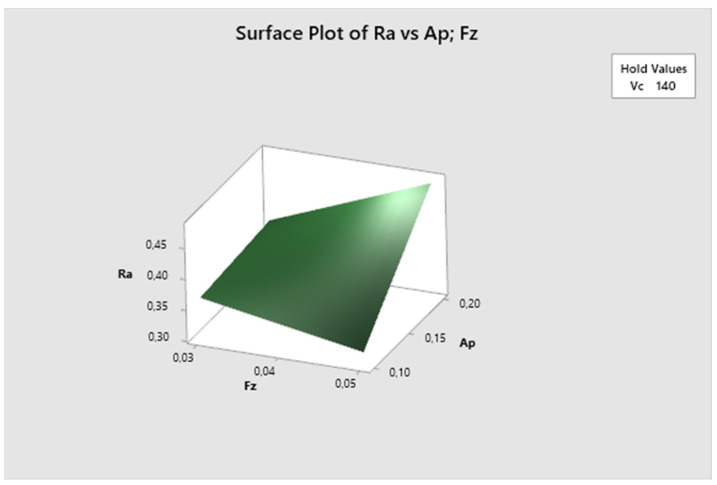
The third experiment-surface plot of Ra—$a_{p}$; $f_{z}$.

Figure 30 displays that the cutting speed parameter is fixed. It is evident that the lowest Ra values are obtained with higher feed per tooth and shallower depth of cut, increasing with the higher depth of cut. The lowest Ra value is observed at $f_{z}$ 0.05 mm/z and $a_{p}$ 0.1 mm.

The conducted study examined the impact of optimized cutting parameters on the efficiency of production and the quality of zirconia dental crowns. Based on the obtained results, it was found that the correct setting of cutting parameters such as cutting speed, feed per tooth, and depth of cut, in conjunction with the appropriate tool selection, leads to a significant reduction in surface roughness to below 0.2 µm, which is crucial for dental applications without the need for further surface polishing. The achieved results are consistent with previous studies [35,41,42,43], which also emphasize the importance of optimizing cutting parameters to achieve high surface quality. Future research should include a broader range of machining conditions and long-term monitoring of the quality and durability of zirconia crowns in clinical practice. In conclusion, our findings highlight the importance of optimizing cutting parameters to improve the quality and efficiency of zirconia dental crown production, and further research should continue to explore and refine these parameters for even better and more consistent results.

A contact profilometer was used for measuring surface roughness, operating on the principle of direct contact between the sensor and the examined surface. Surface irregularities were recorded by the movement of a stylus probe over the sample’s surface. The AFM (Atomic Force Microscopy) method can also be used to characterize material surfaces [44], which records height differences as changes in the force acting on the probe tip. This method is currently primarily employed in the field of nanomeasurement, with a focus on cellular research [45], or nano-engineered implants [46].

The cutting parameters set this way in the machining of zirconia dental crowns influence manufacturing efficiency and product quality. Proper adjustment of parameters such as cutting speed, feed per tooth, and depth of cut leads to a higher quality surface finish of the crowns, reducing surface roughness (Ra) and minimizing surface defects such as microcracks. These cutting parameters also enhance manufacturing efficiency by reducing tool wear, thereby extending tool life and lowering tool replacement costs. The efficient use of cutting parameters shortens production times and increases productivity, allowing for faster production of crowns with consistently high quality. This approach achieves greater precision and reliability in the manufacturing process of zirconia dental crowns, leading to better patient outcomes and higher competitiveness for manufacturers.

The future direction of machining zirconia crowns for dental implants includes several innovations and trends aimed at improving the quality, efficiency, and accuracy of production. In the context of zirconia crown machining, the future direction could focus on highly progressive intelligent machining. This involves the use of machines with adaptive control, which automatically adjust cutting parameters based on real-time machining conditions. As a result, even lower surface roughness values (Ra) could be achieved. Another step in the future could be the use of advanced tools such as diamond tools. The use of diamond tools for machining zirconia can ensure excellent surface quality, as can tools with nanostructured coatings: nanotechnology enables the creation of coatings that increase tool wear resistance and improve the final surface roughness of the machined part.

## 4. Conclusions

The milling of dental materials is currently a highly prevalent technology for producing dental products. The conducted study revealed that the selection of tools and the combination of cutting parameters significantly affect the final surface roughness of dental crowns. Both coated and uncoated tools were used for machining. The results indicate that coated tools with an appropriate tip angle substantially influence the final surface roughness, which greatly impacts the efficiency of manufacturing dental implant crowns. In the first series, an uncoated tool with a 30° tip angle was used. This experiment yielded higher Ra values, which are not very suitable for patients as additional operations, such as final surface polishing, are necessary due to the surface roughness results. In the second series, a tool with a 30° tip angle and X.CEED (AlTiN) coating was used. This experiment achieved the lowest Ra values, which are very suitable for patients since, with parameters v_c_ 150, f_z_ 0.05, and a_p_ 0.1/0.2, all measurements resulted in surface roughness values below 0.2 µm. In the final series, a tool with a 15° tip angle was used, achieving values between 0.22 and 0.56 µm. Based on the conducted study, it can be indicated that the most suitable tool for the specified machining parameters of YML zirconia is a coated tool with an X.CEED (AlTiN) coating and a 30° tip angle. Considering the requirements of this practice, the second series of experiments is the most suitable.

## Figures and Tables

**Table 1:** Material properties [38].

**Physical Properties**	
Chemical composition	ZrO_2_
Volume density	6.05 g/cm^3^
Porosity	0.5> %
**Mechanical Properties**	
Microhardness Vickers	1150 (Hv 0.5)
Young’s modulus	205 GPa
Tensile strength	551 MPa
Elastic modulus	186 GPa
Flexural strength	75 MPa
Compressive strength	3000 MPa
Poisson’s ratio	0.33
Fracture toughness	10 MPa.m^½^
Shear modulus	80 GPa

**Table 2:** Determination of factors in the design of the experiment.

Name	Factor	Unit	−1	+1
X1	$\mathrm{Cutting}\;\mathrm{speed}\;v_{c}$	m/min	130	150
X2	$\mathrm{Feed}\;\mathrm{per}\;\mathrm{tooth}\;f_{z}$	mm/z	0.05	0.07
X3	$\mathrm{Depth}\;\mathrm{of}\;\mathrm{cut}\;a_{p}$	mm	0.1	0.2

**Table 3:** The first experiment—process parameters and measured value.

	$v_{c}$	$f_{z}$	$a_{p}$	Ra
1	130	0.05	0.1	0.22
2	130	0.05	0.2	0.31
3	130	0.07	0.1	0.26
4	130	0.07	0.2	0.21
5	150	0.05	0.1	0.18
6	150	0.05	0.2	0.13
7	150	0.07	0.1	0.24
8	150	0.07	0.2	0.30
9	130	0.05	0.1	0.28
10	130	0.05	0.2	0.41
11	130	0.07	0.1	0.33
12	130	0.07	0.2	0.47
13	150	0.05	0.1	0.21
14	150	0.05	0.2	0.19
15	150	0.07	0.1	0.34
16	150	0.07	0.2	0.41
17	130	0.05	0.1	0.36
18	130	0.05	0.2	0.29
19	130	0.07	0.1	0.45
20	130	0.07	0.2	0.37
21	150	0.05	0.1	0.24
22	150	0.05	0.2	0.19
23	150	0.07	0.1	0.36
24	150	0.07	0.2	0.40

**Table 4:** The second experiment—process parameters and measured values.

	$v_{c}$	$f_{z}$	$a_{p}$	Ra
1	130	0.05	0.1	0.18
2	130	0.05	0.2	0.16
3	130	0.07	0.1	0.15
4	130	0.07	0.2	0.21
5	150	0.05	0.1	0.13
6	150	0.05	0.2	0.11
7	150	0.07	0.1	0.18
8	150	0.07	0.2	0.22
9	130	0.05	0.1	0.19
10	130	0.05	0.2	0.23
11	130	0.07	0.1	0.29
12	130	0.07	0.2	0.26
13	150	0.05	0.1	0.12
14	150	0.05	0.2	0.15
15	150	0.07	0.1	0.20
16	150	0.07	0.2	0.31
17	130	0.05	0.1	0.18
18	130	0.05	0.2	0.27
19	130	0.07	0.1	0.35
20	130	0.07	0.2	0.28
21	150	0.05	0.1	0.17
22	150	0.05	0.2	0.11
23	150	0.07	0.1	0.26
24	150	0.07	0.2	0.22

**Table 5:** The third experiment—process parameters and measured values.

	$v_{c}$	$f_{z}$	$a_{p}$	Ra
1	130	0.05	0.1	0.22
2	130	0.05	0.2	0.31
3	130	0.07	0.1	0.42
4	130	0.07	0.2	0.50
5	150	0.05	0.1	0.36
6	150	0.05	0.2	0.24
7	150	0.07	0.1	0.37
8	150	0.07	0.2	0.56
9	130	0.05	0.1	0.38
10	130	0.05	0.2	0.37
11	130	0.07	0.1	0.41
12	130	0.07	0.2	0.47
13	150	0.05	0.1	0.34
14	150	0.05	0.2	0.28
15	150	0.07	0.1	0.39
16	150	0.07	0.2	0.45
17	130	0.05	0.1	0.44
18	130	0.05	0.2	0.36
19	130	0.07	0.1	0.24
20	130	0.07	0.2	0.56
21	150	0.05	0.1	0.47
22	150	0.05	0.2	0.39
23	150	0.07	0.1	0.34
24	150	0.07	0.2	0.42

## Data Availability

The original contributions presented in the study are included in the article material, further inquiries can be directed to the corresponding author.
